# Adsorption Sequencing as a Rapid Method to Link Environmental Bacteriophages to Hosts

**DOI:** 10.1016/j.isci.2020.101439

**Published:** 2020-08-06

**Authors:** Patrick A. de Jonge, F.A. Bastiaan von Meijenfeldt, Ana Rita Costa, Franklin L. Nobrega, Stan J.J. Brouns, Bas E. Dutilh

**Affiliations:** 1 Theoretical Biology and Bioinformatics, Science4Life, Utrecht University, 3584 CH Utrecht, the Netherlands; 2 Department of Bionanoscience, Kavli Institute of Nanoscience, Delft University of Technology, 2629 HZ Delft, the Netherlands

**Keywords:** Ecology, Environmental Science, Microbiology, Techniques in Genetics

## Abstract

An important viromics challenge is associating bacteriophages to hosts. To address this, we developed adsorption sequencing (AdsorpSeq), a readily implementable method to measure phages that are preferentially adsorbed to specific host cell envelopes. AdsorpSeq thus captures the key initial infection cycle step. Phages are added to cell envelopes, adsorbed phages are isolated through gel electrophoresis, after which adsorbed phage DNA is sequenced and compared with the full virome. Here, we show that AdsorpSeq allows for separation of phages based on receptor-adsorbing capabilities. Next, we applied AdsorpSeq to identify phages in a wastewater virome that adsorb to cell envelopes of nine bacteria, including important pathogens. We detected 26 adsorbed phages including common and rare members of the virome, a minority being related to previously characterized phages. We conclude that AdsorpSeq is an effective new tool for rapid characterization of environmental phage adsorption, with a proof-of-principle application to Gram-negative host cell envelopes.

## Highlights

• AdsorpSeq allows rapid determination of bacteria-bacteriophage interactions
• Model phages can be differentially sequenced based on binding ability with AdsorpSeq
• With AdsorpSeq 26 new phage-host interactions were determined in hospital wastewater

## Graphical Abstract

**Figure:**
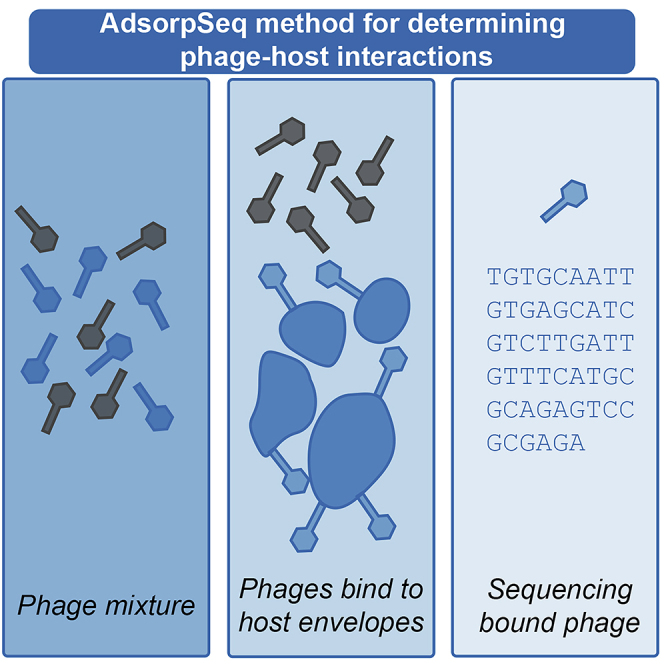

## Introduction

Bacteriophages (viruses that infect bacteria) are omnipresent and impact every ecosystem (Cobián Güemes et al., 2016). Their impact on microbial communities makes phages both useful and detrimental. On the one hand, they are potential bioengineered drug delivery systems (Karimi et al., 2016) and alternatives to antimicrobials (Nobrega et al., 2015). On the other hand, they spread bacterial pathogenicity (Chen and Novick, 2009) and disrupt food production chains like milk fermentations used in the dairy industry (Marcó et al., 2012). Phages also affect ecosystems at larger scales by controlling bacterial evolution and community structure, affecting, e.g., our microbiomes (De Sordi et al., 2019; Manrique et al., 2016), marine nutrient cycling through bacterial lysis (Corinaldesi et al., 2012; Danovaro et al., 2008), and global oxygen production by encoding photosynthesis genes that are expressed in cyanobacterial hosts (Sharon et al., 2009). Although their global importance makes understanding phage-host interactions crucial, most remain undetermined (Cobián Güemes et al., 2016).

A major reason for the mass of undetermined phage-host interactions is a shortage of readily applicable viromics techniques that can simultaneously (1) identify phages in an environmental sample and (2) link them to host(s). Unstudied phage genomes can be identified with metagenomics, but despite constant improvements (e.g., Ahlgren et al., 2017; Galiez et al., 2017; Liu et al., 2019; Mihara et al., 2016; Villarroel et al., 2016; Zhang et al., 2017) it remains challenging to predict to which hosts these phages adsorb, especially at low taxonomic levels (Edwards et al., 2016). Although CRISPR-Cas memory (spacers) and prophage regions can result in reliable host predictions (Edwards et al., 2016), many bacterial lineages do not have CRISPR-Cas systems (Burstein et al., 2016) and not all phages form prophages. Beyond computational approaches, phages can be linked to their host with isolation techniques like double-layer agar plates. Such techniques depend on lytic phages to form visible plaques (Abedon and Yin, 2009; Łoś et al., 2008; Serwer et al., 2007) and can be biased to phages with narrow host ranges (Guyader and Burch, 2008; Kauffman et al., 2018). These assays furthermore often employ a few highly related hosts, each needing a separate assay (Hyman and Abedon, 2010). Thus, available information on phage host range is limited. Other proposed methods include meta3C, which infers interactions based on physical proximity of phage and host DNA (Marbouty et al., 2017), and those summarized in an earlier review (Edwards et al., 2016). However, such methods are generally cumbersome and thereby hard to implement.

An alternative approach to determining phage-host interactions is by focusing on the first step of the phage infection cycle, the adsorption of the phage to bacterial surface receptors. Although phage adsorption is not always followed by successful phage infection, it is a crucial step for successful infections and often specific (de Jonge et al., 2019a). Utilizing phage adsorption specificity could thus allow studies of phage-host interactions in environmental samples. This was recently shown through viral tagging (Deng et al., 2012; Džunková et al., 2019) where fluorescently labeled phages are added to bacteria. Bacteria bound by fluorescently labeled phages are isolated with fluorescence-activated cell sorting, and phage-bacterium pairs are sequenced. This approach allows abundant viruses to be linked to hosts, but it can remain challenging to identify phage-host links for rare members of the virome. Finally, viral tagging requires a specialized experimental setup.

Here, we rapidly identify phage-host pairings by linking cell envelope adsorption to phage sequencing and statistical analysis (adsorption sequencing or AdsorpSeq). AdsorpSeq allows identification of novel phages and their host interactions by exploiting differential migration of phages bound to host receptors and unbound phages in agarose gel electrophoresis. This enables selective sequencing of phages based on their interaction with cell envelopes of a specific host. Thereby multiple phages that interact with a given host can be rapidly and simultaneously identified. We show that model phages can be differentially identified based on the presence of their receptor molecule. Subsequently, we apply AdsorpSeq on a hospital wastewater virome and the cell envelopes of nine taxonomically distinct Gram-negative bacteria, uncovering 26 novel phage-host interactions with a range of abundances in the virome.

## Results and Discussion

### Identification of Model Phages Based on Adsorption to Their Hosts

**Figure 1:**
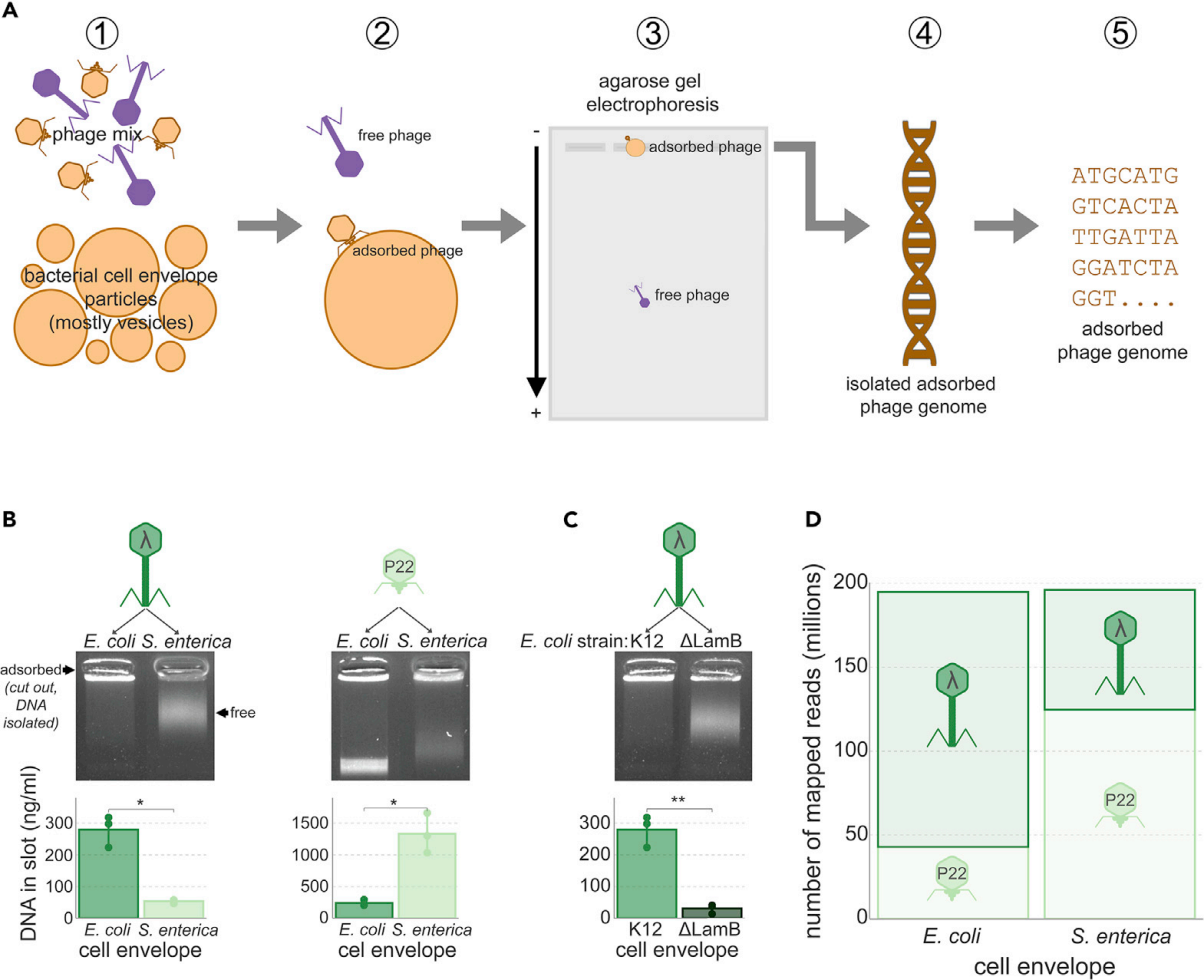
AdsorpSeq Allows the Selective Sequencing of Model Phages Based on Adsorption (A) Schematic of AdsorpSeq. It shows the main steps of (1) mixing phages with bacterial cell envelopes, (2) allowing phages to adsorb to cell envelopes, (3) separating phages using agarose gel electrophoresis based on adsorbing capability, (4) isolating the genomes of adsorbed phages, and (5) sequencing genomes of adsorbed phages isolated from gels. (B) Adsorption of phages λ and P22 to host cell envelopes hinders their migration into agarose gels. Agarose gels of phages λ and P22 after being added to cell envelope suspensions of *E. coli* K12 and *S. enterica* S1400, and bar graphs showing DNA quantities that were isolated from the gel slots at the top of the gels. Arrows indicate the location of free phages (migrated into the gel) and adsorbed phages (in the gel slot) in the first gel. This is identical in the other gels. (C) AdsorpSeq maintains receptor molecule specificity of phage λ. Agarose gel of phage λ after being added to *E. coli* strain K12, to which it can adsorb, and *E. coli* ΔLamB, to which it cannot adsorb. Bar graph depicts DNA isolated from gel slots at the top of the gel. Note: although the smear seems visually stronger in the K12 lane, significantly more DNA was retained in the well containing the K12 envelope fraction than in the ΔLamB envelope fraction (see bar graphs). (D) Applying AdsorpSeq to a mixture of phages leads to differentiation based on adsorbing capacity. Stacked bar graph showing the number of reads mapped to phages λ and P22 after AdsorpSeq was applied using an equal mixture of the two phages and cell envelopes from either *E. coli* K12 or *S. enterica* S1400. Significance levels according to a paired t test, error bars depict standard deviations, points are biological replicates. ∗p < 0.05, ∗∗p < 0.01.

AdsorpSeq aims to selectively sequence phages based on their adsorption to bacterial cell envelopes. This is achieved by a five-step process (steps 1–5 in Figure 1A). First, a phage mixture is added to a cell envelope suspension that was isolated from a bacterium of interest (step 1 in Figure 1A). An incubation then allows phage adsorption to their receptors (2). Next, agarose gel electrophoresis separates bound and unbound phages (3). Unlike unbound phages, phages bound to cell envelope suspensions will migrate slower into agarose gels owing to the larger size and altered charge of the adsorption complex. The result is rapid separation of phages based on adsorption abilities. Finally, genomic material of bound phages is isolated from the gel (4) and sequenced (5). To validate AdsorpSeq, we tested the method with *Escherichia* phage λ and *Salmonella* phage P22 as two model phages with well-described adsorption properties. These two phages differ in host, morphology, and the receptor type. The receptor of *Siphoviridae* phage λ is the *E. coli* maltose pore protein LamB (Wang et al., 2000), whereas the *S. enterica* subspecies *enterica* (hereafter: *S. enterica*) lipopolysaccharide O-antigen chain serves as receptor for *Podoviridae* phage P22 (Andres et al., 2010). Because absorption to the bacterial cell envelope does not guarantee successful infection (de Jonge et al., 2019a) AdsorpSeq may detect phage-bacteria interactions beyond infecting host range. In addition, the isolation of bacterial membrane fractions may alter the interactions of the viruses with these membranes. For example, membrane fragments can form vesicles both in normal and inverted conformations (Coakley et al., 1977; Poole, 1993), therewith supplying the phage with the ability to bind to the cell envelope side that is normally pointed inward. However, as both phages are specific to their respective receptors (Andres et al., 2010; Randall-Hazelbauer and Shwartz, 1973), we could gauge preservation of adsorption specificity in AdsorpSeq.

As a first test, we added either phage λ or P22 to *E. coli* and *S. enterica* cell envelope suspensions. Upon adding non-host cell envelopes (e.g., adding *S. enterica* to λ), phage particles migrated into agarose gels, whereas adding host cell envelopes (e.g., adding *E. coli* to λ) resulted in phage particle retention around the sample slot at the origin of electrophoresis (Figure 1B). These gel regions consequently contained significantly more DNA when using host cell envelopes than when using non-host cell envelopes (two-tailed t test, p < 0.05). This showed that adsorption of phage particles to host cell envelopes prevented migration into agarose gels. To confirm receptor specificity, we repeated the experiment with a LamB-knockout strain (ΔLamB) (Baba et al., 2006), which resulted in phage λ losing adsorption ability (Figure 1C). These results agree with earlier studies (Andres et al., 2010) showing slower migration of phage particles into agarose gels after addition of purified phage receptor particles.

After establishing that presence of host cell envelopes alters phage migration in agarose gels, we used this property to preferentially sequence phage genomes from a mixture based on the presence of host cell envelopes. We performed AdsorpSeq with a mixture of equal parts phage λ and P22, which we added to cell envelopes of either *E. coli* or *S. enterica*. Upon sequencing of genomic material isolated from agarose slices, two to three times more reads mapped to the phage λ genome than the P22 genome when *E. coli* cell envelopes were added and vice versa (Figure 1D). The resulting difference in phage genome abundance was highly significant (Fisher's exact test, p = 2.5 × 10^−16^). As AdsorpSeq was thus capable of discerning phage-host associations in a simple phage mixture, we next applied it to a complex environmental phage mixture.

### AdsorpSeq Results in Selection of Unique Phage Subsets

We next applied AdsorpSeq to identify phages targeting specific bacterial cell envelopes in a complex virome derived from a hospital wastewater influent pipe (Figure S1A). In such environments, phages can be found at concentrations of 10^8^–10^10^ particles per milliliter (Otawa et al., 2007; Rosario et al., 2009; Tamaki et al., 2012; Wu and Liu, 2009). Phage adsorption targets consisted of cell envelope suspensions from nine taxonomically diverse Gram-negative bacteria, including three *Enterobacterales* (*Escherichia coli, Citrobacter freundii,* and *Klebsiella pneumoniae*), two *Pseudomonadales* (*Pseudomonas aeruginosa* and *Acinetobacter baumannii*), two *Bacteroidales* (*Bacteroides fragilis* and *Bacteroides dorei*), one *Burkholderiales* (*Ralstonia pickettii*), and one *Fusobacterales* (*Fusobacterium necrophorum*). All these bacteria are either part of the healthy human gut microbiome (e.g., *B. dorei*) (Huttenhower et al., 2012) or pathogens linked with hospital infections (e.g., *K. pneumoniae*) (Rice, 2008). We expect that AdsorpSeq may be most useful for Gram-negative bacteria, because these bacteria possess an outer membrane that is both easily isolated and contains the main receptors targeted during phage infections (Nobrega et al., 2018; Silva et al., 2016). Phage receptors in Gram-positive bacteria are often associated with the thick outer peptidoglycan layer (Dowah and Clokie, 2018) that can be difficult to break and isolate. Thus, in the following validation and application of AdsorpSeq, we focused our efforts on Gram-negative bacteria. Next to the virome treated with the cell envelopes of these nine bacteria, we sequenced the full untreated virome as control. As we increased DNA quantities of all samples by multiple displacement amplification (MDA), which alters apparent viral community compositions (Pinard et al., 2006), the full virome was sequenced both before and after MDA. This allowed us to gauge and correct for MDA effects during data analysis (below).

Sequencing of the nine cell envelope-treated samples and the two viromes (pre- and post-MDA) resulted in 138 Mbp of read-level data. A cross-assembly resulted in 23,373 contigs longer than 2,500 bp, representing 71.4% of the total dataset, as determined by mapping the reads back to the contigs (for annotated contig metadata and contig abundances, see Table S1). Taxonomic classification showed that the cross-assembly contained 1,111 viral contigs and a further 8,921 contigs that were taxonomically unclassified, whereas the remaining 13,341 contigs were mostly derived from bacteria, with a minority of archaeal and eukaryote contigs. The group of unclassified contigs likely reflects the large numbers of unstudied human gut phages (Shkoporov and Hill, 2019). We therefore combined the viral and unclassified sets to create a dataset of 10,032 confirmed and suspected viral contigs, which represented 51.5% of total reads. The percentage of reads represented by selected contigs fluctuated across the cell envelope-treated samples, ranging from 41.0% (*E. coli* treated) to 81.9% (*B. fragilis* treated) of reads (median: 70.2%, Figure S1B). These differences between the samples were a first indication that phage adsorption depended on the cell envelope suspension used.

Although there were 842 contigs with identical ends in the selected dataset representing putatively complete and circular genomes, many contigs also represented likely genomic fragments. We therefore assigned contigs with similar tetranucleotide usage patterns and read depth patterns across the nine cell envelope-treated samples to 1,058 viral populations. Binning of contigs is based on nucleotide usage and abundance signals. Thus, the bins do not necessarily represent single phage genomes, but rather groups of contigs with similar characteristics, which we refer to as viral populations. Some viral populations may contain fragments of different phages specifically binding to a host and similar phages that non-specifically bind to the same host. Jaccard distances based on viral population relative abundances showed distinct dissimilarity between the cell envelope-treated samples (Figure S1C). This indicated that each sample contained a unique set of viral populations and thus selected different phages. The viral populations with the highest abundance across the samples were also highly abundant in both virome controls (Figures S2A and S2B) suggesting that some phage particles are retained in the wells through non-specific interactions, as also observed for λ and P22 (Figure 1D). Perhaps some phages are physically prevented from migrating into agarose gels by the vesicles that cell envelope suspensions likely form (Poole, 1993). Although we did not achieve perfect separation between bound and unbound phage particles, we concluded from the evident dissimilarity in composition between the samples (Figure S1C) that selection of phages by AdsorpSeq is dependent on the type of bacterial cell envelope used.

### Selection of Phages with Putative Adsorption Activity

To identify phages that specifically adsorb to one or more of the nine bacterial species, we selected viral populations that were overrepresented in one or more of the samples. To focus on the strongest adsorbing phages first, we defined overrepresented viral populations based on outlier analysis (see Methods for details), which meant that we selected viral populations with a relative abundance of at least 1.58 times higher in one sample than in the other eight samples. Relative abundance values from samples of the same bacterial taxonomic order were discounted when determining overrepresented viral populations to allow for phages with a broad adsorption or infection host range. In total, 123 viral populations represented phages that specifically adsorb to the cell envelope fractions of one of the nine bacterial species (Figure S3A).

We next refined the selection of putatively adsorbing viral populations by applying two filters. The first filter removed viral populations that were positively selected for by MDA. MDA can result in efficient rolling circle amplification but has also been shown to lead to a bias for small single-stranded DNA (ssDNA) phage genomes (Kim and Bae, 2011; Probst et al., 2015; Yilmaz et al., 2010). This held true in our dataset, as comparing viral populations in the virome before and after MDA showed 10- to 100-fold higher amplification of ssDNA *Microviridae* than other phage families (Figure S3B). Note that 23,564 reads from the unamplified virome mapped to *Microviridae* contigs, showing that these small circular DNA viruses are abundant in the hospital virome and their detection does not fully depend on MDA. To reduce the impact of MDA bias, we thus filtered out 79 viral populations with strong MDA selection, leaving 44 viral populations that passed the MDA selection filter (Figure S3A).

In addition to MDA selection, we tested if certain phages were universally selected for by the AdsorpSeq technique (Figure S3C). This identified 18 putative adsorbing viral populations for which the relative abundance in all nine samples was higher than in post-MDA virome (Figure S3A). This methodological bias was highest among *Inoviridae* and *Microviridae* (Figure S3C). Although *in silico* evidence suggests that some phages may have very broad host ranges (Paez-Espino et al., 2016; Roux et al., 2016), most phages likely have a narrow host range spanning a few closely related strains within the same species or genus (de Jonge et al., 2019a; Džunková et al., 2019). We thus interpreted our findings as a methodological selection bias that may reflect the inability of a phage particle to migrate into agarose gels owing to large size, low charge, or non-specific interactions with bacterial cell envelopes. This may explain the stronger methodological selection pressure on *Inoviridae*, which have lipid membrane-adsorbing coat proteins (Stopar et al., 2003). The 18 viral populations that were under strong methodological bias were filtered out of the final selection of adsorbing vial populations.

After applying MDA and methodological selection filters, 26 viral populations with predicted adsorbing activity remained (Figure S3A). All 26 selected viral populations were highly specific to cell envelopes of a single bacterium and thus represented phages with a single predicted host. This was despite our allowance of broad host range at the order level in selecting viral populations but agrees with a recent report that found that broad host-range phages are rare in gut viromes based on single-cell viral tagging experiments (Džunková et al., 2019).

Notably, 22 of the 26 selected viral populations putatively adsorb to *Proteobacteria* cell envelopes, consistent with recent findings that *Proteobacteria* are the dominant bacterial phylum in global wastewater treatment plant microbiomes and are abundant in wastewater influent (Petrovich et al., 2019; Wu et al., 2019). It is thus likely that *Proteobacteria* phages are common in wastewater microbiomes, which supports our findings.

### Selected Viral Populations Are Rare and Similar to *Proteobacteria* Phages

**Figure 2:**
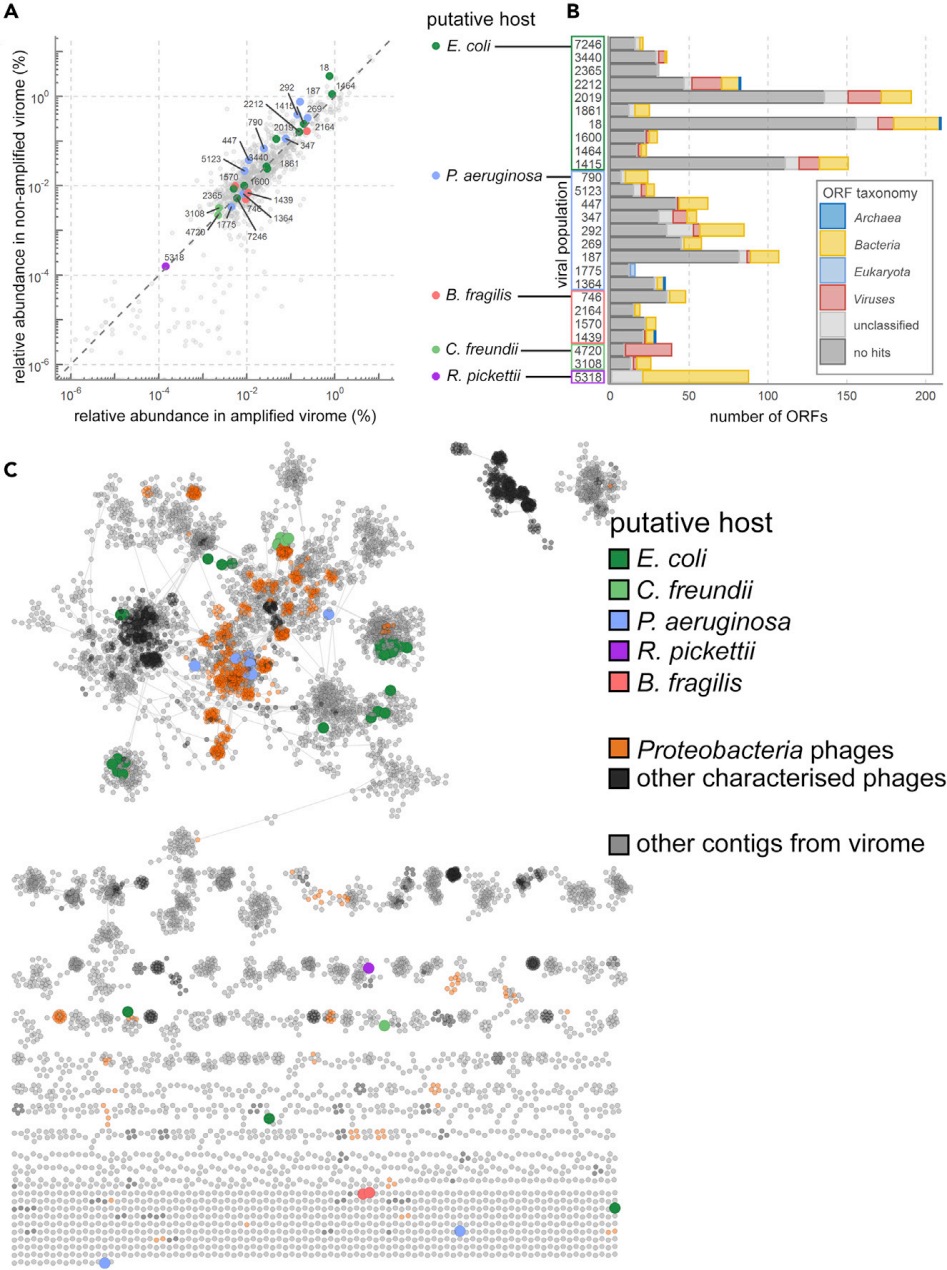
Most Selected Viral Populations Represent Rare and Uncharacterized Viral Sequences (A) Relative abundance of selected viral populations with adsorption predictions in the virome before and after MDA shows that AdsorpSeq is not biased for abundant or rare phage sequences. Numbers next to data points show the viral population number. (B) ORF-level taxonomical predictions using CAT show most ORFs from selected viral populations have no similarities in the NCBI nr protein sequence database (dark gray). Some contigs had database hits but could not be classified because the hits involved proteins from different superkingdoms. These are labeled as unclassified (light gray). (C) The hospital wastewater virome contained a large diversity of uncharacterized phage sequences, as shown by a gene-sharing network of 10,032 viral contigs and all phage genomes in the NCBI viral RefSeq database (Pruitt et al., 2007), made using vContact2 (Bin Jang et al., 2019). Large colored contigs represent those in the final selection of 26 putative adsorbing viral populations. *Proteobacteria*-infecting characterized phages are orange.

Next, we examined the final selection of 26 viral populations with adsorption predictions. Their relative abundance in the virome ranged from 0.0001% to 1%, covering the spectrum from relatively rare to relatively abundant in the virome before and after MDA (Figure 2A). Characterization of the ORFs by direct homology searches showed that the majority of ORFs (64% of total) in selected viral populations had no significant similarity to protein sequences in the National Institute for Biotechnology Information (NCBI) non-redundant (nr) database (Agarwala et al., 2017) (Figure 2B). From these findings we concluded that AdsorpSeq can be used to identify adsorption hosts of both common and rare uncharacterized environmental phages.

To assess AdsorpSeq host predictions, we performed a whole genome clustering of all viral populations (including those without adsorption predictions) with all characterized phages whose genome sequences were available in the NCBI bacterial and viral RefSeq V85 database (Pruitt et al., 2007) (Figure 2C). First, we observed that the contigs of the viral populations identified with AdsorpSeq clustered together, confirming our contig binning approach. Second, most of the 22 selected viral populations that were predicted to adsorb to *Proteobacteria* species were similar to characterized *Proteobacteria* phages. This supports the notion that the viral populations that were detected with AdsorpSeq on the cell envelopes of specific bacterial pathogens reflect naturally occurring phages capable of infecting these bacteria.

### Selected Viral Populations with Similarity to Characterized Phages

To further assess the phage-host associations detected by AdsorpSeq, we searched for matches between contigs in selected viral populations and bacterial CRISPR-Cas spacers. We identified 1.4 million spacers predicted from bacteria in the Pathosystems Resource Integration Center (PATRIC) database (Wattam et al., 2014) and queried them against contigs in the selected viral populations. No full-length identical protospacers were detected on any of the viral contigs. Two contigs from viral populations adsorbing to *Pseudomonas aeruginosa* contained spacer hits with a single mismatch each. These hits originated from CRISPR-Cas arrays encoded on the genomes of *Aeromonas caviae* and *Tolumonas auensis,* which are members of the same taxonomical class as *P. aeruginosa* (*Gammaproteobacteria*). Several examples have been observed of phage genera that adsorb to differently related bacteria, such as Tequatroviruses (Nolan et al., 2006), Gap227likeviruses (Wang et al., 2016), and Plpelikeviruses (Comeau et al., 2012; Walker et al., 2019). Alternatively, cell envelope adsorption does not necessitate successful infection, as many factors play a role in the completion of the infection cycle (de Jonge et al., 2019a), including differences in transcriptional programs between hosts (Howard-Varona et al., 2018) or the presence of molecular defense systems (Hampton et al., 2020; Labrie et al., 2010). No other selected viral populations contained contigs with spacer hits with fewer than five mismatches. As five mismatches corresponds to a ∼50% false discovery rate at the species level (Edwards et al., 2016), hits with more mismatches were not considered for analysis.

**Figure 3:**
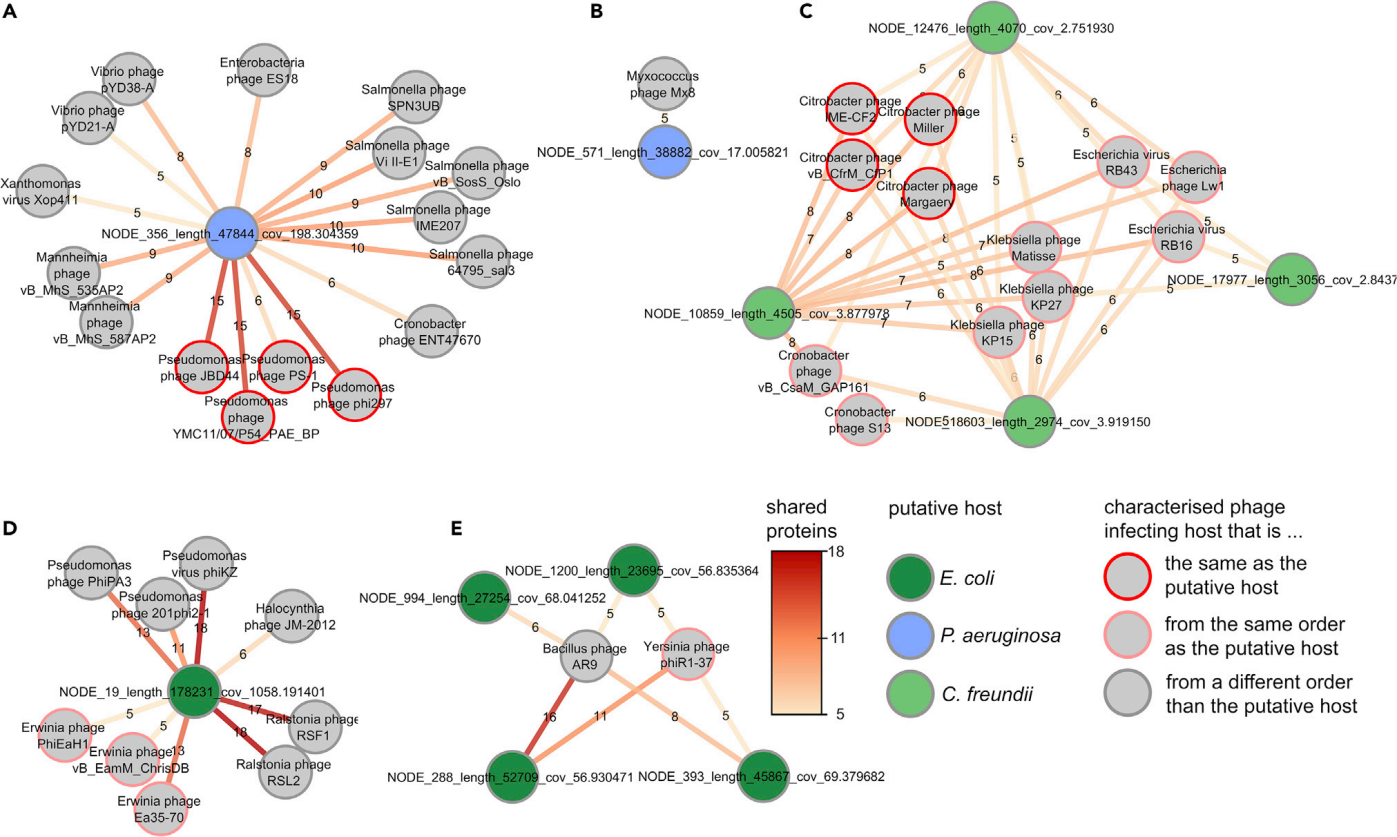
Selected Viral Populations Related to Characterized Phages Protein-sharing networks of viral populations show their relationships to characterized phages. ORFs from selected viral populations were used in BLASTp searches against proteins of phages in the viral RefSeq database (Pruitt et al., 2007). Bubbles are phages. Edge color and labels show similar protein counts (E-value ≤ 10^−5^). (A) *P. aeruginosa*-adsorbing viral population 292. (B) *P. aeruginosa*-adsorbing viral population 447. (C) *C. freundii*-adsorbing viral population 4720. One additional contig did not share protein similarity to characterized phages. (D) *E. coli*-adsorbing viral population 18. (E) *E. coli*-adsorbing viral population 2019.

Next, we placed the viral populations identified with AdsorpSeq in the context of characterized phages by using protein sharing networks. We narrowed our search to contigs that contained over five ORFs with known homologs, yielding five selected viral populations with extensive similarity to characterized phages (Figure 3 and Table S3).

**Figure 4:**
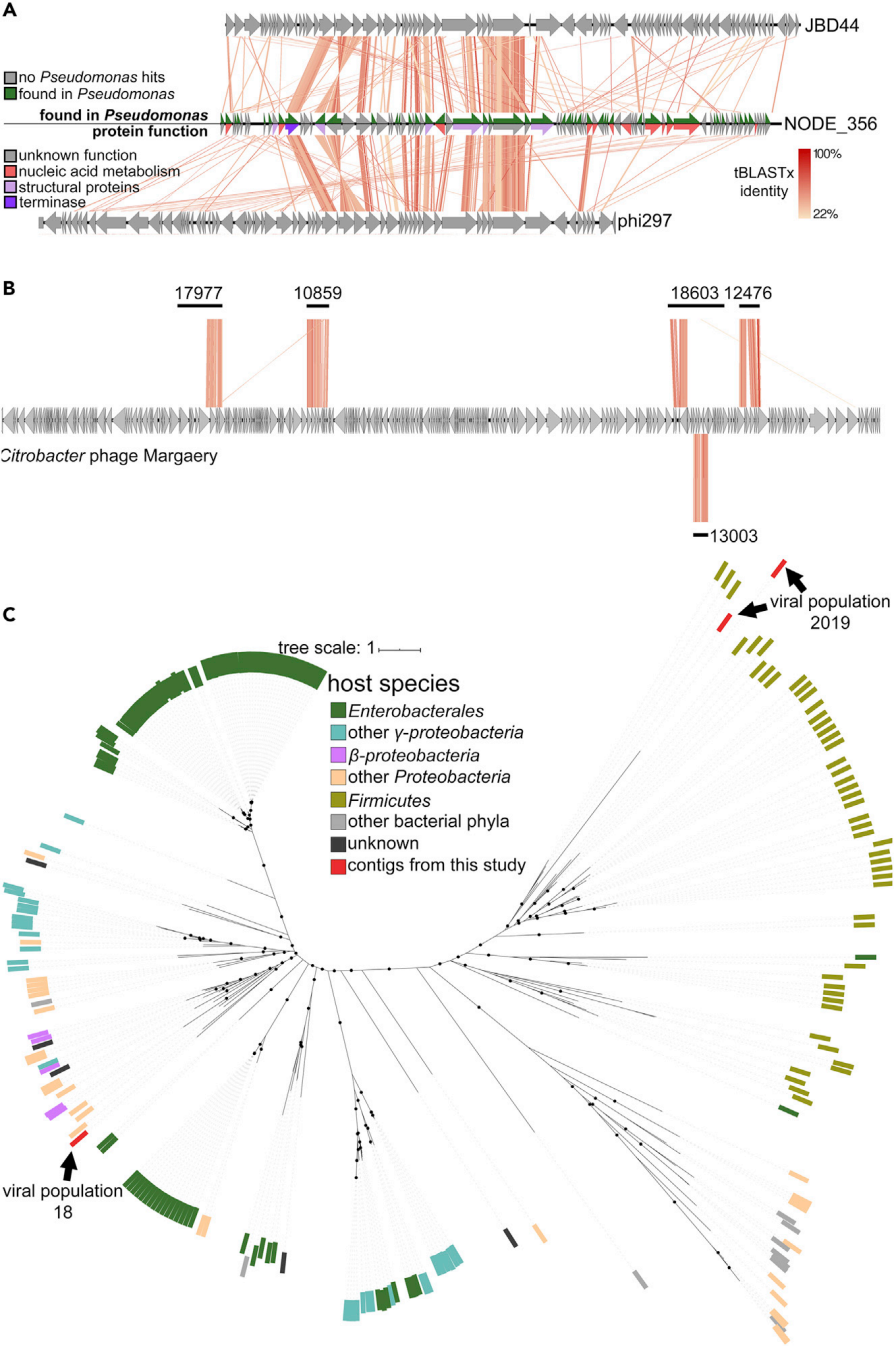
Several Viral Populations and their Relations to Characterized Families (A) Similarity of viral population 447, containing contig 356, to *Pseudomonas* phages JBD44 and phi297. Depicted is a whole genome comparison made using Easyfig (Sullivan et al., 2011). In the line representing contig 356, the top half shows ORFs with BLASTp hit against *Pseudomonas* bacteria proteins in the NCBI nr database, whereas the bottom half shows protein function. (B) Similarity between five contigs from *C. freundii*-adsorbing viral population 4720 and T4-like *Citrobacter* phage Margaery, as shown by genome comparisons made using Easyfig (Sullivan et al., 2011). Numbers indicate contig numbers, contig 13,003 was placed below phage *Margaery* as it overlaps with contig 18,603. Colors indicate tBLASTx hits and use the same legend as (A). (C) The relation of *E. coli*-adsorbing viral populations 18 and 2019 to jumbo phages displayed in an unrooted approximate maximum likelihood tree of jumbo phage terminases. Dots on branches represent ultrafast bootstrap support of ≥85 (Hoang et al., 2018).

First, *P. aeruginosa*-adsorbing viral population 292 contained a single circular contig on which 15/85 ORFs (17.6%) were similar to proteins from the known *Pseudomonas* phages JBD44, phi297, and YMC11/07/P54_PAE_BP (Figure 3A). Notably, phi297 infects the *P. aeruginosa* strain used in this study (Bourkal'tseva et al., 2011). The fifteen shared proteins included the often conserved terminase (Low et al., 2019; Serwer et al., 2004) and several adjacent genes (Figure 4A and Table S4), stressing the relatedness between these phages. Following earlier practice (Lavigne et al., 2008), this protein similarity would classify these phages into the same taxonomical family. Although the conservative CAT ORF-level predictions provided no taxonomic classifications for most ORFs in viral population 292, direct homology searches found significant similarity (BLASTp, e-value ≤ 10^−5^) to proteins from *Pseudomonas* bacteria for 45 of the 85 ORFs (53%, Figure 4A and Table S4) from viral population 292. This viral population thus represents a novel *Pseudomonas* phage identified in the hospital sewage inlet by AdsorpSeq with *P. aeruginosa* cell envelopes as bait.

A second viral population that also adsorbed to *P. aeruginosa* (447) contained a contig of 38,882 bp that had 5/62 ORFs with similarity to *Myxococcus* phage Mx8 (Figure 3B), which translates to less than 5% of 85 Mx8 ORFs. The similarity between viral population 447 and phage Mx8 is thus limited to a small number of genes, which, although not adjacent, all belong to the structural section of the Mx8 genome (Table S3). We suggest that these limited shared proteins reflect shared gene cassettes (Hatfull and Hendrix, 2011; Lavigne et al., 2008).

In addition to the *Pseudomonas-*adsorbing viral populations, viral population 4720 represents a *Citrobacter*-adsorbing phage. This viral population contains five short (2,974–4,505 bp) contigs. The four longest of these had significant protein sequence similarity (BLASTp, e-value ≤ 10^−5^) to at least five ORFs from several *Citrobacter* phages (Figure 3C), whereas all five contigs showed full-length sequence similarity to *Citrobacter* phage Margaery (tBLASTx, e-value ≤ 0.001, Figure 4B). Combined, they shared similarity to 25/280 (9%) *Citrobacter* phage Margaery proteins and several other T4-like *Citrobacter* phages (Figure 4B). As these known *Citrobacter* phages have genomes over 150,000 bp, we suggest that viral population 4720 may represent fragments of a larger *Citrobacter* phage genome.

Finally, two selected *E. coli*-adsorbing viral populations (18 and 2019) shared five to eighteen ORFs with several known jumbo phages (Figures 3D and 3E). These included *Yersinia* phage phiR1-37 and *Erwinia* phage Ea35-70, both of which infect bacteria from the same taxonomic order as *E. coli* (*Gammaproteobacteria*). Notably, *E. coli* phages like T4 (Tétart et al., 1996) and T3 (Garcia et al., 2003) need only limited genomic alterations to extend their host range to *Yersinia* species. To gain added insight into the relation of these phages to other jumbo phages, we built a phylogeny of their terminase genes (Figure 4C). Earlier analysis showed that jumbo phages with phylogenetically closely related terminases often infect related hosts (Yuan and Gao, 2017). For our analysis, we gathered 148 sequences from the NCBI nr database (Agarwala et al., 2017) and 74 jumbo phage proteins from a recent study (Al-Shayeb et al., 2020) that had significant homology to viral population 18 and 2019 terminases (BLASTp, e-value ≤ 10^−5^). The resulting tree placed viral population 2019 in a branch that mostly held recently described jumbo phages (Al-Shayeb et al., 2020), with hosts predicted to belong to the *Firmicutes*. Viral population 18 terminase belonged to a more diverse clade with multiple *Ralstonia* phages, a single *Alteromonadaceae* bacterial sequence (a *γ-proteobacteria* species), and multiple jumbo phages previously predicted to infect proteobacterial hosts (Al-Shayeb et al., 2020). These phages might interact with a surface element that is common to all these hosts. This hypothesis is reinforced by the fact that *Ralstonia* were long assigned to the genus *Pseudomonas* within the γ-proteobacteria, despite differences in membrane composition (Yabuuchi et al., 1995) and lipopolysaccharide structure (Zdorovenko et al., 2008). Alternatively, in similar fashion to *Enterobacteria* phage phi92 (Schwarzer et al., 2012), they may harbor multiple receptor-adsorbing proteins in their large genomes. Besides the terminase, the circular contig in viral population 18 notably also contained an FtsZ homolog. In *Pseudomonas* jumbo phages, FtsZ-proteins are part of a nucleus-like defense mechanism (Chaikeeratisak et al., 2017; Mendoza et al., 2019), together with a nucleus-forming protein. Interestingly, protein homology searches did not identify similar nucleus-forming proteins in viral population 18, although this population consists of a single circular contig. It may thus contain a yet unknown system like the nucleus-like defense mechanism found in *Pseudomonas* jumbo phages or use the FtsZ homolog in a system that is different altogether.

The results discussed above represent 6 of 26 AdsorpSeq-selected viral populations with at least five protein similarities to characterized phages. The ORFs encoded on the remaining 20 selected viral populations represented novel or highly divergent proteins, unrelated to previously characterized phages. Together, our results underscore the ability of AdsorpSeq to uncover environmental phages and their potential host associations, without bias for known or abundant phages.

### Conclusion

Despite recent advances in viromics, linking environmental phage sequences to hosts remains problematic (Edwards et al., 2016). Here, we presented AdsorpSeq, a rapid method for detecting phage adsorption to host cellular envelopes. AdsorpSeq is easily implementable, as besides sequencing it uses only commonly available laboratory methods, such as cell disruption and agarose gel electrophoresis.

In the current study, we validated AdsorpSeq on model bacteriophages. This showed that AdsorpSeq can separate phages based on the presence of a specific host receptor in bacterial cell envelopes. Future improvements to AdsorpSeq could exploit this feature, for instance, through heterologous expression of certain bacterial features of interest and uncovering phages that adsorb to them. Therewith, AdsorpSeq could aid in uncovering interactions between phages and microbial surface molecules like antimicrobial efflux pumps. AdsorpSeq uncouples analysis of phage adsorption and phage infection, potentially allowing for analysis of phage-bacterium interactions that do not result in infection, for instance, owing to intracellular defense systems (Hampton et al., 2020).

The experiments laid out in this study identified 26 phage-host interactions in a hospital wastewater virome. Most of these were predictions of phages adsorbing to *Proteobacteria,* which is consistent with recent findings that these bacteria are ubiquitous and abundant in global waste water communities (Petrovich et al., 2019; Wu et al., 2019). Several of the putative adsorbing viral populations represented rare members of the sampled hospital wastewater virome. Although rare here, these viruses may still be important in another time and place as they become transiently dominant through ecological dynamics (Arkhipova et al., 2018). Moreover, expanding our knowledge to include rare members of the virosphere is important to address fundamental questions about the evolution of viral genes and genomes (de Jonge et al., 2019b; Mavrich and Hatfull, 2017), to identify candidate viruses with potentially promising genomic properties for phage therapy (Nobrega et al., 2015) and for targeted monitoring (Metsky et al., 2019). This underscores the necessity for novel methods such as AdsorpSeq with which phage-host interactions can be rapidly assessed, even for uncommon phages within a complex environmental mixture.

### Limitations of the Study

Here we presented AdsorpSeq as a method to link environmental bacteriophages to their bacterial hosts. An important caveat for future AdsorpSeq applications is that it determined whether phages bind to the cell surface of a host cell, which is not necessarily followed by a successful infection. Investigations on the ability of phages with AdsorpSeq binding predictions to complete infections, such as phage isolations, would thus be a useful addition of the method. Of course, targeted phage isolation is a non-trivial task, and an inability to observe infection in methods such as plaque assays does not necessarily indicate an inability to infect, as phage infections can be (pseudo-)lysogenic or chronic and as not all lytic phages form plaques (Serwer et al., 2007). Additionally, we focused our efforts on Gram-negative bacterial envelopes, whereas the applicability of AdsorpSeq to Gram-positive bacteria remains to be tested.

### Resource Availability

#### Lead Contact

Further information and requests for resources and reagents should be directed to and will be fulfilled by the Lead Contact, Bas E. Dutilh (bedutilh@gmail.com)

#### Materials Availability

Materials and protocols used in this study are available from the authors upon request. This study did not generate new unique reagents.

#### Data and Code Availability

The accession number for the sequencing data project reported in this paper is ENA: PRJEB37817. The accession number of sequencing reads reported in this paper are ENA: ERS4427880–ERS4427890, whereas the accession number of cross-assembled contigs reported in this paper is ENA: ERZ1305919.

## Methods

All methods can be found in the accompanying Transparent Methods supplemental file.

## Supplemental Information

Document S1. Transparent Methods and Figures S1–S4

Table S1. Read Depth Data of All Cross-Assemble Contigs, Related to Figure 1

Table S2. CAT Classifications of ORFs in Selected Viral Populations, Related to Figures 3 and 4

Table S3. BLASTp Hits to characterized Phages Among Selected Contigs, Figure 3 and 4

Table S4. Annotation of Viral Population 292, Related to Figure 3
