# Supplementary material for: Adsorption Sequencing as a Rapid Method to Link Environmental Bacteriophages to Hosts
Source: iScience. 2020 Aug 6;23(9):101439. doi: 10.1016/j.isci.2020.101439 (PMC7452251; doi:10.1016/j.isci.2020.101439)
Supplement: Document S1. Transparent Methods and Figures S1–S4 [file mmc1.pdf]

iScience, Volume *23*

Supplemental Information

Adsorption Sequencing

as a Rapid Method to Link

Environmental Bacteriophages to Hosts

Patrick A. de Jonge, F.A. Bastiaan von Meijenfeldt, Ana Rita Costa, Franklin L. Nobrega, Stan J.J. Brouns, and Bas E. Dutilh

Transparent methods

General data reporting

All metagenomics tools used default parameters, except where explicitly stated otherwise. All graphs were plotted using the ggplot (Gómez-Rubio, 2017) v3.2.1 package in R. All chemicals were obtained from Sigma-Aldrich, unless explicitly stated otherwise.

Data availability

All genomic data has been uploaded to the European Nucleotide Archive (ENA) under project PRJEB37817. Reads are available under ENA accession numbers ERS4427880-ERS4427890, while the cross-assembled contigs are available under ENA accession number ERZ1305919.

Bacterial cultivation and phage stock preparation

We tested the validity of AdsorpSeq with two model phages and their hosts. These were *Escherichia* phage λ (DSMZ #4499) infecting *Escherichia coli* K12 BW25113 (DSMZ #27469) and *Salmonella* phage P22 (DSM #18523), infecting *Salmonella enterica* subsp. *enterica* serovar Enteritidis S1400/94 (hereafter called *Salmonella enterica*) (Allen-Vercoe et al., 1997). For additional tests of phage λ specificity, we used *E. coli* JW3996 (ΔLamB) from the Keio strain collection (Baba et al., 2006), which lacks the λ protein receptor (Wang et al., 2000). For subsequent AdsorpSeq application on a hospital wastewater virome, we used nine bacterial strains. These were *Acinetobacter baumannii* (DSMZ #300007), *Klebsiella pneumoniae* (ATCC #11296), *Ralstonia pickettii* (DSMZ #6297), *Pseudomonas aeruginosa* PA01 (DSMZ #22644), *Fusobacterium necrophorum* D12, *Citrobacter freundii* 4_7_47CFAA, *Escherichia coli* 4_1_47FAA , *Bacteroides fragilis* 3_1_12, and *Bacteroides dorei* 5_1_36/D4. The latter five strains were provided by the reference catalogue of the Human Microbiome Project (HMP) from the University of Guelph in Guelph, Canada. All bacteria, except for the obligate anaerobic *Bacteroides* and *Fusobacterium* strains, were aerobically cultivated in lysogeny broth (LB) at 37°C while under agitation. The obligate anaerobic strains were cultivated at 37°C in anaerobic Columbia broth. This medium was made anaerobic by boiling and cooling under a stream of nitrogen gas, followed by dispensation in bottles which were closed with rubber stoppers and aluminium crimp caps. Bottles were subjected to three rounds of vacuum and nitrogen gas and autoclaved at 121°C for 20 minutes.

Phage λ and P22 stocks were produced with the soft-agar overlay method as described (Kutter, 2009). To determine phage titres, an aliquot of 0.1 ml exponentially growing bacterial culture was added to 5 ml 0.7% (w/v) LB agarose. This mixture was layered on top of 1.5% (w/v) LB agar and allowed to dry. Phage stock was diluted in a series of 10-fold dilutions using SM buffer (100 mM NaCl, 8 mM $MgSO_4$ x 7 H_2O, 50 mM Tris-HCl pH 7.5) and 10 µl of each dilution was placed on the plates. After 16 h incubation at 37°C, plaques were counted.

Bacterial cell envelope isolations

To isolate bacterial cell envelopes, bacteria were first grown overnight (aerobic) or for 3 days (anaerobic) as described under “bacterial cultivation and phage stock preparation”. The resulting cultures were centrifuged at 10,000 x *g*, 4°C for 15 minutes. Supernatant was discarded and cell pellets were washed with the original volume of lysis buffer (50 mM Tris HCl pH 7.5, 2 mM $MgCl_2$). This was centrifuged again, the supernatant was discarded, and the pellet was re-suspended in 4 volumes of lysis buffer per gram wet cell weight with the addition of 1 tablet of cOmplete EDTA-free protease inhibitor. Cells were lysed by thrice passing the suspension through a model CF1 Cell Disruptor (Constant Systems) at 1.5 kBar. After removal of cell debris by centrifugation at 12,000 x *g*, 4°C for 15 minutes, cell envelopes were collected by centrifugation at 225,000 x *g* ,4°C for 1 hour. The supernatant was discarded, cell envelope pellets were re-suspended in lysis buffer and centrifuged again. Soluble proteins were removed by resuspension of the cell envelope pellet in 200 mM NaCl + 20 mM Tris HCl, pH 7.5 and a third centrifugation. Supernatant was removed and cell envelope pellets were dissolved in 20 mM Tris HCl pH 7.5 + 200 mM NaCl at a concentration of 10 mg/ml and stored at -20°C until further use.

Agarose assays with model phages

AdsorpSeq was first tested using phage λ and P22 and bacterial cell envelope suspensions from their hosts. Equal volumes of 10^{11} pfu/ml phage titre and 10 mg/ml cell envelope suspension were mixed. To allow phages to adsorb to bacterial cell envelope suspensions, the mixture was incubated at room temperature for 20 minutes. Bound and unbound phages were separated by applying the mixtures on a 1% (w/v) agarose gel and applying a current of 20 V/cm for 20 minutes using a Mupid One gel electrophoresis system (Eurogentec). The slots at the top of the gel, which contain bound phages, were cut out of the gel using a fresh scalpel knife and DNA was isolated using a Zymoclean gel DNA recovery kit (Zymo Research). The recovered DNA was quantified with a Qubit dsDNA HS assay kit and Qubit fluorometer (Thermo Fisher Scientific).

Hospital virome preparation

Material for the virome consisted of two litres of wastewater influent kindly provided by the Reinier de Graaf hospital wastewater treatment facility in Delft, the Netherlands. To remove debris and cellular material, the material was filtered in stages using coffee filters, 0.45 µm filters, and 0.2 µm filters. This may have selected against giant bacteriophages. Subsequently the virome was concentrated to 70 ml (i.e. approximately 30 times) using a Vivaflow tangential flow filter with a 100 kDa cut-off (Sartorius). The sample was stored at 4ºC until further use.

To isolate phage DNA from the virome, viral capsids were first broken by incubating a 1 ml virome aliquot with 10 µg/ml proteinase K and 0.2 % SDS at 56ºC for 1 hour. Afterward, DNA was purified by phase separations using consecutively phenol, phenol/chloroform, and chloroform. The aqueous phase was collected and 0.1 volume 3 M sodium acetate (pH 5.2) and 2.5 volumes ice-cold absolute ethanol were added. The mixture was incubated overnight at -20ºC and DNA was pelleted by centrifugation at 21,000 x *g* for 15 minutes at 4ºC. The pellet was washed with one volume of 70% ethanol, and re-pelleted by repeating the centrifugation. The DNA pellet was dissolved in TE buffer (1 mM EDTA, 10 mM Tris HCl pH 8) and the DNA concentration was ascertained using a Qubit dsDNA HS assay kit and Qubit fluorometer (Thermo Fisher Scientific).

Sample preparation and sequencing

Two sequencing experiments were performed. The first was a confirmation that AdsorpSeq can be used with a mixture of phage λ and P22, while the second applied AdsorpSeq to the hospital wastewater virome and nine bacterial strains (see "bacterial cultivation and phage stock preparation"). For the first sequencing experiment, phage λ and P22 were mixed at a titre of approximately $1 \cdot 10^8$ pfu/ml each. Incubation with cell envelopes of either *E. coli* BW25113 or *S. enterica* and DNA isolation were performed as described under "agarose assays with model phages". For the second sequencing experiment, the same methodology was used on samples prepared from the hospital wastewater virome and cell envelope preparations. To increase DNA quantities, the samples were subjected to multiple displacement amplification (MDA) using an Illustra genomiphi v3 φ29 polymerase kit (GE Lifesciences) according to the instructions. Primers were removed from the amplified samples using AMpure XP beads (Beckman Coulter) and the final amplicons were eluted in 50 µl of TE buffer. DNA concentrations were determined using a Qubit dsDNA HS assay kit and Qubit fluorometer (Thermo Fisher Scientific). All samples with cell envelope suspensions added to them were prepared in biological quadruplicates. The whole procedure was repeated for the

samples including both cell envelopes and viromes, for a total of two technical duplicates that each consisted of biological quadruplicates. Virome-only controls only had biological duplicates. For both experiments, these virome-only controls consisted of DNA isolated from the phages both before and after MDA.

Library preparation and sequencing of all samples was performed at the Utrecht sequencing facility (USEQ) in Utrecht, the Netherlands. Libraries were prepared using the TruSeq DNA nano kit (Illumina), and samples were sequenced on a NextSeq500 run with 2x150 bp paired reads (Illumina).

Sequencing data analysis

To increase the read quality from our sequencing experiments, Illumina reads were quality trimmed, poly-G tails were removed, and remaining adapters were removed using fastp v0.20.0 (Chen et al., 2018) (options -g, -x). For the application of AdsorpSeq on the hospital waste water virome, trimmed reads of the unamplified virome and all bacterial samples were cross-assembled into 1,013,501 contigs using metaSPAdes v3.11.0 (Nurk et al., 2017). Reads from all samples were mapped to the genomes of the model phages and their hosts (model phage experiment) or the cross-assembled contigs (hospital waste water virome experiment) using the Burrows-Wheeler Aligner v0.7.12-r1039 (Li and Durbin, 2009). The number of reads that mapped to each genome or contig were determined using the idxstats tool in samtools v1.454 (Li et al., 2009).

For the experiment using hospital waste water, mean read depths of each contig in each sample were determined using the JGI summarize bam contig depths tool in metaBAT v2.12.1 (Kang et al., 2015). Additionally, all reads from the amplified virome were mapped against the contigs to determine which contigs were selected for by the amplification procedure. Contigs belonging to the host genomes were identified by a megaBLAST search against the host genomes using BLAST v2.6.0+ (Camacho et al., 2009). The 12,496 contigs with over 50% coverage and over 50% identity were removed as host contigs. Subsequently, contigs were taxonomically annotated using the contig annotation tool (CAT) v5.0.3 (Von Meijenfeldt et al., 2019), which uses homology searches of ORFs against the National Centre for Biotechnology Information (NCBI) non-redundant protein database (nr) (Agarwala et al., 2017) to predict contig taxonomy. CAT used prodigal v2.6.3 (Hyatt et al., 2010) to predict ORFs. Contigs with a superkingdom classification of “Viruses” and a score of 1 (which indicates that all predicted ORFs in a contig were classified in that superkingdom), those that had other superkingdom classifications with scores below 1, and those that could not be classified at all were selected for further analysis. This resulted in a dataset of 13,032 (putative) viral contigs. Putatively completed circular contigs were identified using a custom script that checked

whether the start and end of a contig contained identical sequences, following earlier studies (Jahn et al., 2019; Roux et al., 2017).

We binned the selected contigs according to their tetranucleotide usage patterns and their read depth patterns across the nine bacterial samples using metaBAT v2.12.1 (Kang et al., 2015). Because viral genomes are smaller than the bacterial genomes for which metaBat was originally built, we lowered the minimal contig length allowance to 2,500 bp and the minimum bin size to 10,000 bp. In addition, we decreased the minimum mean coverage necessary in each sample for binning to 0.001 to allow for contigs that might not be present in all samples (i.e. options -m 2500, -s 10000, and -x 0.001). Contigs that metaBAT could not bin, whether due to low coverage or short length, were discarded. We binned genomic fragments into viral populations (Gregory et al., 2019) based on similarity in tetranucleotide usage and abundance patterns by using metaBat resulting in 1158 viral populations containing a total of 6,572 contigs.

Selection of overrepresented viral populations

To select putative cell envelope-adsorbing viral populations, the abundance per viral population in each sample was calculated with the following formula:

$$\text{abundance}_{\text{viral population}} = \frac{\sum\left(\text{read depth}_{\text{viral population}} \cdot \text{contig length}_{\text{viral population}}\right)}{\sum \text{contig length}_{\text{viral population}}} \qquad \text{(Equation 1)}$$

This abundance was then divided by the total abundance of all viral populations in the sample and expressed in a percentage to obtain the relative abundance for each viral population in each sample. For each viral population, the highest relative abundance across all cell envelope-treated samples was divided by the next highest relative abundance from a sample that used cell envelope suspensions from different taxonomical order. Positive outliers among the fractions between the top two samples of all viral populations were determined with the following formula:

$$\text{outlier} \geq 75\%_{\text{all viral populations}} + \text{interquartile range}_{\text{all viral populations}} \cdot 1.5 \qquad \text{(Equation 2)}$$

The boundary for inclusion as putative adsorbing viral population established by this was 1.58 (Supplementary Figure 4A). A total of 123 putative adsorbing viral populations that constituted as outliers were selected.

MDA and methodological selection filters

To determine the extent that viral populations were selected for by MDA, we divided the relative abundance of each viral population in the post-MDA virome by that in the pre-MDA virome. To ascertain the relationship between phage taxonomy and MDA selection factor, we plotted the

values of all viral populations with a CAT classification at the family level that belonged to a phage lineage. From the resulting MDA selection factors, positive outliers were determined using equation 2. The 79 viral populations with MDA selection factors above 3.8, as defined by equation 2, were discounted (Supplementary Figure 4B).

In addition to the MDA selection factor, we also determined which viral populations were universally selected for by AdsorpSeq. For this, we calculated a methodological selection factor by dividing the relative abundance of each viral population per sample by the relative abundance in the amplified virome. As a result, each viral population had one methodological selection factor for each cell envelope-treated sample for a total of nine per viral population. After determining positive outliers with equation 2, the 18 viral populations which were above the outlier threshold of 3.43 in all nine samples were removed from the dataset (Supplementary Figure 4C). After accounting for these two biases, our dataset contained 26 putatively adsorbing viral populations which combined contained 83 contigs.

<u>Analysis of putatively adsorbing viral populations</u>

To uncover the extent of viral 'dark matter' among the putatively adsorbing viral populations, we plotted the ORF-specific taxonomical classifications obtained with CAT (Von Meijenfeldt et al., 2019) of all ORFs found in putative adsorbing viral populations. While CAT is built to classify contigs, it first predicts ORF-specific taxonomy and subsequently uses these results to predict contig taxonomy. Relatedness of the putative adsorbing viral populations and characterised phages was established by performing a BLASTp all vs all on a dataset composed of all ORFs from putative adsorbing viral populations and all ORFs from characterised phages in the NCBI bacterial and viral RefSeq V85 database (Pruitt et al., 2007). BLASTp searches were performed using diamond v0.9.29.130 (Buchfink et al., 2014), with a bit score significance cut-off of 50. Links between viral populations and characterised phages were displayed using Cytoscape v3.7.2 (Shannon et al., 2003). For further analysis of the links between putative adsorbing viral populations and known phages, we performed a taxonomical assignment on all selected contigs using vContact2 v.0.9.8 (Bin Jang et al., 2019), while the resulting network was visualised using Cytoscape v3.7.2 (Shannon et al., 2003).

Contigs from putative adsorbing viral populations were annotated using PROKKA v1.11 [80], with the metagenomic option enabled and with both the viral and bacterial settings (i.e. options--metagenome and both --kingdom Bacteria and --kingdom Viruses). In addition, distant homologs to ORFs were identified by performing searches against prokaryotic viral orthologous groups (pVOGs) (Grazziotin et al., 2017) using hmmsearch v3.1b2 (Eddy, 2011). To compare contig sequences to characterised phage genomes they were aligned using

Easyfig v2.2.3 (Sullivan et al., 2011), which used BLAST v2.9.0+ (Camacho et al., 2009) to perform tBLASTx searches.

CRISPR-Cas spacer analysis

To search for CRISPR-Cas spacer hits against the putative adsorbing viral populations, spacers were identified in all bacteria Pathosystems Resource Integration Centre (PATRIC) database (Wattam et al., 2014) (accessed February 2019) using detect 2.2.1. This resulted in a dataset of 1,473,418 spacers. These spacers were used as query against contigs from putative adsorbing viral populations in a BLASTn with the option for short sequences enabled (option -task blastn-short) in BLAST v2.9.0+ (Camacho et al., 2009). Only spacer hits with fewer than five mismatches were taken into consideration. In practice, there were no spacer hits with between one and five mismatches.

Jumbo phage terminase phylogeny

Some of the putative adsorbing viral populations contained (fragments of) jumbo phage genomes. It was previously shown that in a terminase phylogenetic tree these phages largely clustered according to host (Yuan and Gao, 2017). Three terminases that we identified in the putative adsorbing viral populations were used as query for a BLASTp search against the NCBI nr-database using the NCBI webserver (Johnson et al., 2008) on January 14 2020. In addition, the three terminases were used as query for a BLASTp search against jumbo phage proteins from Al-Shayeb *et al* (Al-Shayeb et al., 2020). All 222 total hits above a bit score threshold of 50 were combined with the three queries. Duplicated sequences were removed and the remainder was aligned using Clustal Omega v1.2.1 (Sievers and Higgins, 2018). Aligned positions that consisted of more than 90% gaps were trimmed with trimAl v1.2 (Capella-Gutiérrez et al., 2009) (option -gt 0.1). A maximum likelihood tree was constructed using IQ-Tree v1.6.12 (Nguyen et al., 2015) using model finder (Kalyaanamoorthy et al., 2017) and performing 1000 iterations of both SH-like approximate likelihood ratio test and the ultrafast bootstrap approximation (UFBoot) (Hoang et al., 2018). In addition, ten iterations of the tree were separately constructed, as has been recommended (Zhou et al., 2018) (IQ-Tree options -bb 1000, -alrt 1000, and --runs 10). The iteration with the best log-likelihood score, for which model finder selected LG+F+R6, was used for analysis. The tree was visualised using interactive Tree of Life v5.5 (Letunic and Bork, 2019). In addition, we selected the putative FtsZ homolog from viral population 18 (ORF 17) and used it to query the NCBI nr-database using the NCBI webserver (Johnson et al., 2008) on January 14 2020. This did not result in significant hits.

Supplementary figures

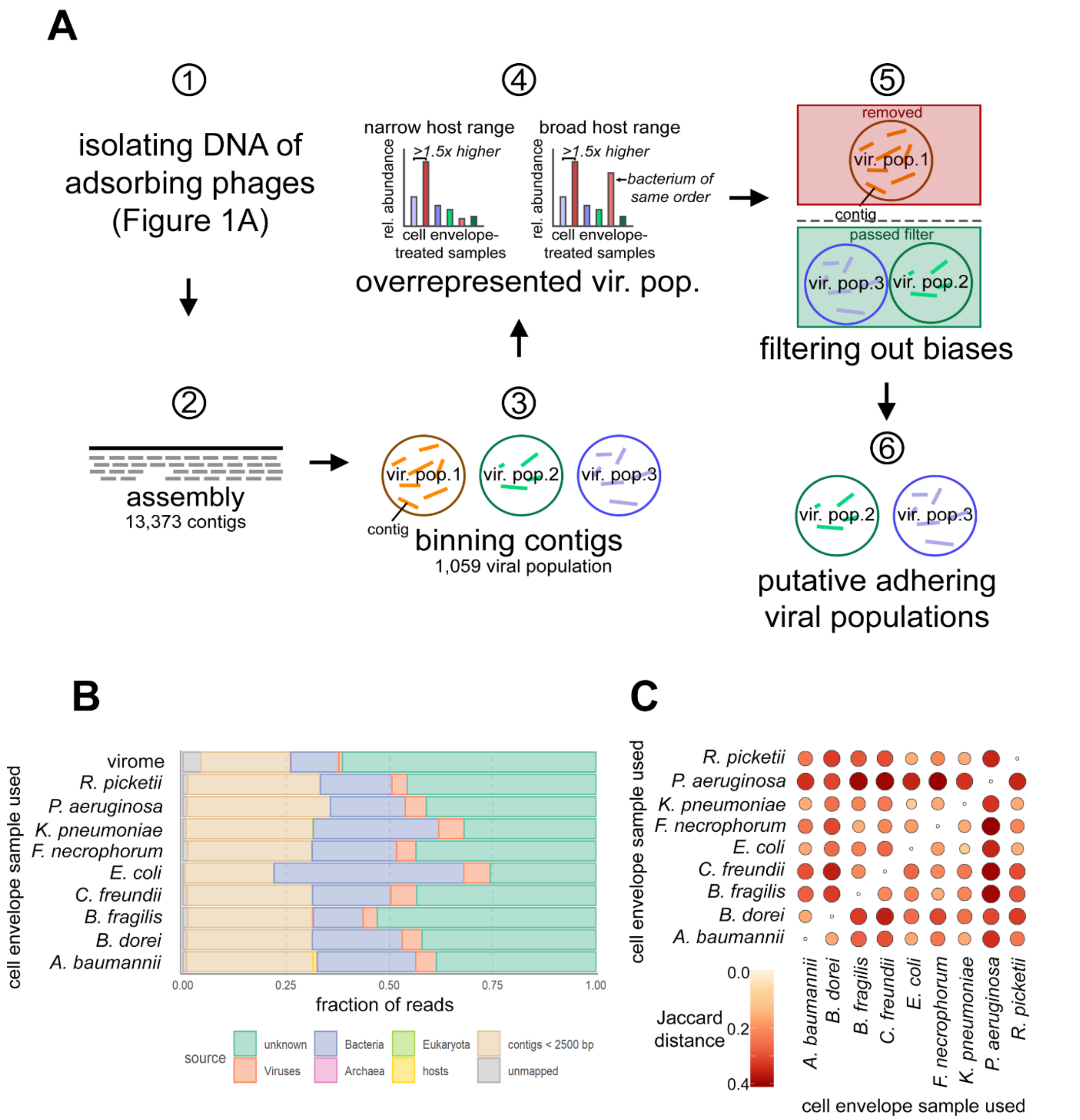

Supplementary Figure 1: Application of AdsorpSeq to a hospital wastewater virome, related to Figure 1 and 2.

(A) Schematic of the approach taken to apply AdsorpSeq to an environmental sample. Step (1) consists of the process depicted in Figure 1A. Subsequently, data analysis consisted of (2) assembly of read-level data into contigs, (3) binning contigs into viral populations based on their relative abundance across the samples, (4) selection of viral populations that were overrepresented in one sample, (5) remove viral populations under multiple displacement amplification (MDA) or methodological bias, and (6) the final selection of viral populations with putative adhering capabilities.

(B) For contigs over 2500 bp, a majority of reads across the all but one of the samples map to unknown or viral contigs. Stacked bar charts of the fragment of reads from each sample that are unmapped, map to short contigs below 2,500 bp, map to one of the nine bacterial hosts, map to contigs that are classifiable at the superkingdom level, and map to unknown/unclassifiable contigs.

(C) Selection of phages by AdsorpSeq is dependent on the species of bacterial cell envelope that is used. A correlogram of the Jaccard distance between the membrane-treated samples, based on 1,058 viral populations. Higher Jaccard distance means greater dissimilarity. Size and colour indicated Jaccard dissimilarity.

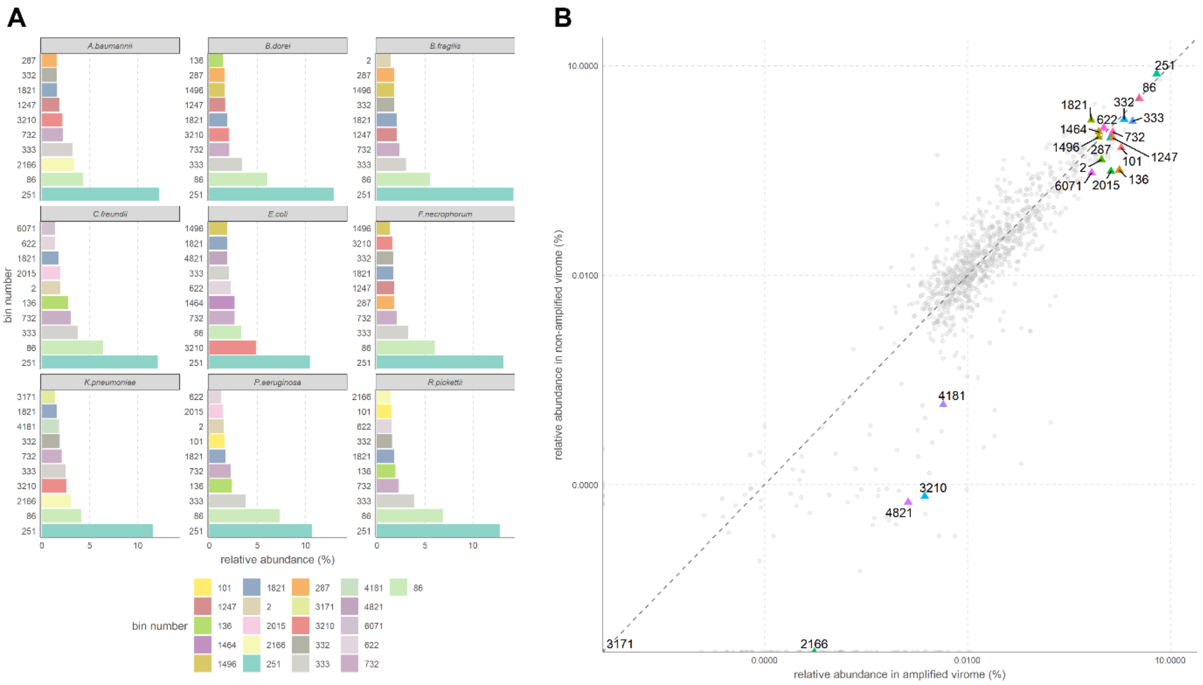

Supplementary Figure 2: The most abundant genome viral populations across the cell envelope-treated samples are similar, related to Figure 2.

(A) Bar charts showing the ten most abundant genome viral populations in each of the nine cell envelope-treated samples and their relative abundances.

(B) The relative abundances of the genome viral populations depicted in (A) in the virome before (y-axis) and after (x-axis) multiple displacement amplification. Numerical labels refer to the genome viral populations.

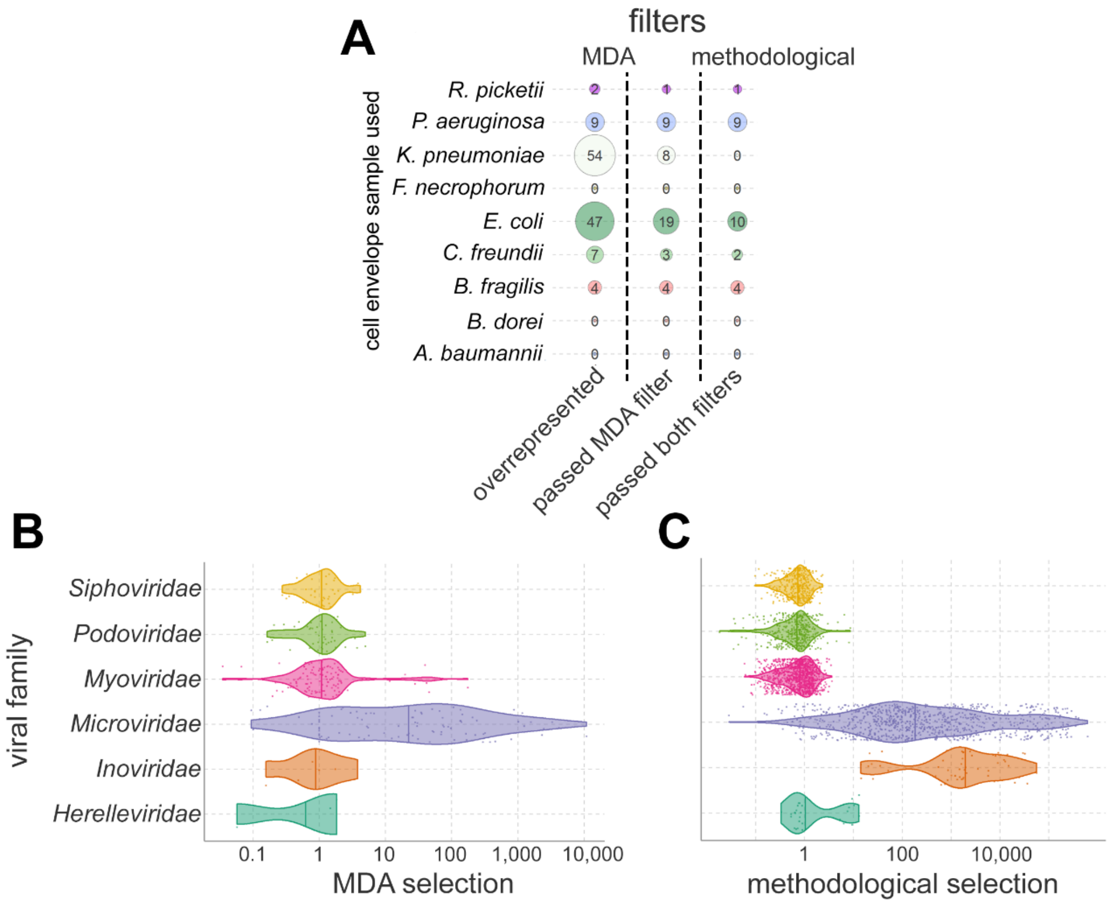

Supplementary Figure 3: Selection of putative adhering viral populations from a hospital wastewater virome, related to Figure 2 and 3.

(A) After filtering for MDA and methodological selection, 26 viral populations with putative adsorbing capacity were selected from the virome. Bubble area shows number of viral populations after sequentially selecting based on being overrepresented in one sample, filtering for MDA selection, and filtering for methodological selection. The third column shows the selected viral populations with putative adhesion activity.

(B) *Microviridae* are highly selected for by MDA, as shown by viral populations with positive CAT predictions within a bacteriophage family. MDA selection is defined as the ratio in relative abundance between the virome before and after MDA. Points in the charts represent individual viral populations.

(C) *Inoviridae* and *Microviridae* undergo high methodological selection, which is defined by the ratio between the relative abundance of a viral population in each sample and in the amplified virome. As there were nine cell envelope-treated samples, each viral population is represented in the plot by nine data points.

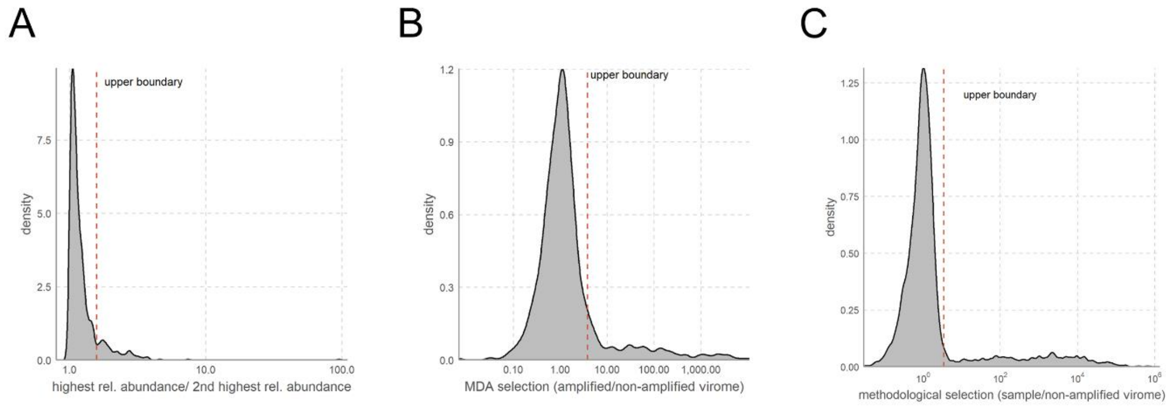

Supplementary Figure 4: Distributions used in selection of viral populations that represent adhering viral populations, related to Figure 2.

(A) Distribution of the ratios between the cell envelope-treated sample with the highest and second highest relative abundance among the viral populations. All viral populations with a ratio above the red dashed line labelled “upper boundary” were selected as being overrepresented in a sample.

(B) Distribution of the ratios in relative abundance between the virome before and after multiple displacement amplification (MDA). All viral populations with a ratio above the red dashed line labelled “upper boundary” were under strong MDA selection.

(C) Distribution of the ratios between the relative abundances in the samples and in the non-amplified virome for each viral population. Each viral population is represented with nine datapoint in this plot; one for each cell envelope-treated sample. All viral populations with a ratio above the red dashed line labelled “upper boundary” were under strong methodological selection.

Supplementary references

Agarwala, R., Barrett, T., Beck, J., Benson, D.A., Bollin, C., Bolton, E., Bourexis, D., Brister, J.R., Bryant, S.H., Canese, K., et al., (2017). Database Resources of the National Center for Biotechnology Information. Nucleic Acids Res. 45, D12–D17.

Al-Shayeb, B., Sachdeva, R., Chen, L.X., Ward, F., Munk, P., Devoto, A., Castelle, C.J., Olm, M.R., Bouma-Gregson, K., Amano, Y., et al., (2020). Clades of huge phages from across Earth’s ecosystems. Nature 578, 425–431.

Allen-Vercoe, E., Dibb-Fuller, M., Thorns, C.J., Woodward, M.J., (1997). SEF17 fimbriae are essential for the convoluted colonial morphology of Salmonella enteritidis. FEMS Microbiol. Lett. 153, 33–42.

Baba, T., Ara, T., Hasegawa, M., Takai, Y., Okumura, Y., Baba, M., Datsenko, K.A., Tomita, M., Wanner, B.L., Mori, H., (2006). Construction of Escherichia coli K-12 in-frame, single-gene knockout mutants: the Keio collection. Mol. Syst. Biol. 2, 2006.0008.

Bin Jang, H., Bolduc, B., Zablocki, O., Kuhn, J.H., Roux, S., Adriaenssens, E.M., Brister, J.R., Kropinski, A.M., Krupovic, M., Lavigne, R., et al., (2019). Taxonomic assignment of uncultivated prokaryotic virus genomes is enabled by gene-sharing networks. Nat. Biotechnol.

37, 632–639.
Buchfink, B., Xie, C., Huson, D.H., (2014). Fast and sensitive protein alignment using DIAMOND. Nat. Methods 12, 59–60.
Camacho, C., Coulouris, G., Avagyan, V., Ma, N., Papadopoulos, J., Bealer, K., Madden, T.L., (2009). BLAST+: Architecture and applications. BMC Bioinformatics 10, 1–9.
Capella-Gutiérrez, S., Silla-Martínez, J.M., Gabaldón, T., (2009). trimAl: A tool for automated alignment trimming in large-scale phylogenetic analyses. Bioinformatics 25, 1972–1973.
Chen, S., Zhou, Y., Chen, Y., Gu, J., (2018). Fastp: An ultra-fast all-in-one FASTQ preprocessor. Bioinformatics 34, i884–i890.
Eddy, S.R., (2011). Accelerated profile HMM searches. PLoS Comput. Biol. 7.
Gómez-Rubio, V., (2017). ggplot2 - Elegant Graphics for Data Analysis (2nd Edition) . J. Stat. Softw. 77, 3–5.
Grazziotin, A.L., Koonin, E. V., Kristensen, D.M., (2017). Prokaryotic Virus Orthologous Groups (pVOGs): A resource for comparative genomics and protein family annotation. Nucleic Acids Res. 45, D491–D498.
Gregory, A.C., Zayed, A.A., Conceição-Neto, N., Temperton, B., Bolduc, B., Alberti, A., Ardyna, M., Arkhipova, K., Carmichael, M., Cruaud, C., et al., (2019). Marine DNA Viral Macro- and Microdiversity from Pole to Pole. Cell 177, 1109-1123.e14.
Hoang, D.T., Chernomor, O., von Haeseler, A., Minh, B.Q., Vinh, L.S., (2018). UFBoot2: Improving the Ultrafast Bootstrap Approximation. Molecular biology and evolution. Mol. Biol. Evol. 35, 518–522.
Hyatt, D., Chen, G.L., LoCascio, P.F., Land, M.L., Larimer, F.W., Hauser, L.J., (2010). Prodigal: Prokaryotic gene recognition and translation initiation site identification. BMC Bioinformatics 11.
Jahn, M.T., Arkhipova, K., Markert, S.M., Stigloher, C., Lachnit, T., Pita, L., Kupczok, A., Ribes, M., Stengel, S.T., Rosenstiel, P., et al., (2019). A Phage Protein Aids Bacterial Symbionts in Eukaryote Immune Evasion. Cell Host Microbe 26, 542-550.e5.
Johnson, M., Zaretskaya, I., Raytselis, Y., Merezhuk, Y., McGinnis, S., Madden, T.L., (2008). NCBI BLAST: a better web interface. Nucleic Acids Res. 36, 5–9.
Kalyaanamoorthy, S., Minh, B.Q., Wong, T.K.F., von Haeseler, A., Jermiin, L.S., (2017). ModelFinder: fast model selection for accurate phylogenetic estimates. Nat. Methods 14, 587–589.
Kang, D.D., Froula, J., Egan, R., Wang, Z., (2015). MetaBAT, an efficient tool for accurately reconstructing single genomes from complex microbial communities. PeerJ 2015, 1–15.
Kutter, E., (2009). Phage host range and efficiency of plating. Methods Mol. Biol., Methods in Molecular Biology 501, 141–9.
Letunic, I., Bork, P., (2019). Interactive Tree Of Life (iTOL) v4: recent updates and new

developments. Nucleic Acids Res. 47, W256–W259.
Li, H., Durbin, R., (2009). Fast and accurate short read alignment with Burrows-Wheeler transform. Bioinformatics 25, 1754–1760.
Li, H., Handsaker, B., Wysoker, A., Fennell, T., Ruan, J., Homer, N., Marth, G., Abecasis, G., Durbin, R., (2009). The Sequence Alignment/Map format and SAMtools. Bioinformatics 25, 2078–2079.
Nguyen, L.T., Schmidt, H.A., Von Haeseler, A., Minh, B.Q., (2015). IQ-TREE: A fast and effective stochastic algorithm for estimating maximum-likelihood phylogenies. Mol. Biol. Evol. 32, 268–274.
Nurk, S., Meleshko, D., Korobeynikov, A., Pevzner, P.A., (2017). MetaSPAdes: A new versatile metagenomic assembler. Genome Res. 27, 824–834.
Pruitt, K.D., Tatusova, T., Maglott, D.R., (2007). NCBI reference sequences (RefSeq): A curated non-redundant sequence database of genomes, transcripts and proteins. Nucleic Acids Res. 35, 61–65.
Roux, S., Emerson, J.B., Eloe-Fadrosh, E.A., Sullivan, M.B., (2017). Benchmarking viromics: An in silico evaluation of metagenome-enabled estimates of viral community composition and diversity. PeerJ 2017, 1–26.
Shannon, P., Markiel, A., Ozier, O., Baliga, N.S., Wang, J.T., Ramage, D., Amin, N., Schwikowski, B., Ideker, T., (2003). Cytoscape: a software environment for integrated models of biomolecular interaction networks. Genome Res. 13, 2498–504.
Sievers, F., Higgins, D.G., (2018). Clustal Omega for making accurate alignments of many protein sequences. Protein Sci. 27, 135–145.
Sullivan, M.J., Petty, N.K., Beatson, S.A., (2011). Easyfig: A genome comparison visualizer. Bioinformatics 27, 1009–1010.
Von Meijenfeldt, F.A.B., Arkhipova, K., Cambuy, D.D., Coutinho, F.H., Dutilh, B.E., (2019). Robust taxonomic classification of uncharted microbial sequences and bins with CAT and BAT. Genome Biol. 20, 1–14.
Wang, J., Hofnung, M., Charbit, A., (2000). The C-terminal portion of the tail fiber protein of bacteriophage lambda is responsible for binding to LamB, its receptor at the surface of Escherichia coli K-12. J. Bacteriol. 182, 508–512.
Wattam, A.R., Abraham, D., Dalay, O., Disz, T.L., Driscoll, T., Gabbard, J.L., Gillespie, J.J., Gough, R., Hix, D., Kenyon, R., et al., (2014). PATRIC, the bacterial bioinformatics database and analysis resource. Nucleic Acids Res. 42, 581–591.
Yuan, Y., Gao, M., (2017). Jumbo bacteriophages: An overview. Front. Microbiol. 8, 1–9.
Zhou, X., Shen, X.X., Hittinger, C.T., Rokas, A., (2018). Evaluating fast maximum likelihood-based phylogenetic programs using empirical phylogenomic data sets. Mol. Biol. Evol. 35, 486–503.
